# Utilizing Ni(II) complex for metal drug-gel particles in cervical cancer treatment and designing novel drugs through machine learning methods

**DOI:** 10.1038/s41598-024-55897-7

**Published:** 2024-03-05

**Authors:** Meiping Jiang, Ruiping Wu, Dongqin Liu, Xiaoli Wang

**Affiliations:** Departments of Radiotherapy, Tumor Hospital of Yunnan Province, The Third Affiliated Hospital of Kunming Medical University, Kunming, Yunnan China

**Keywords:** Coordination polymer, Hydrogels, Cervical cancer, Machine learning, Biochemistry, Bioinorganic chemistry

## Abstract

In the present study, a novel coordination polymer (CP) based on Ni(II), namely, [Ni(L)(D-CAM)(H_2_O)]_n_ (**1**) (H_2_D-CAM = (1*R*,3*S*)-1,2,2-trimethylcyclopentane-1,3-dicarboxylic acid and L = 3,6-bis(benzimidazol-1-yl)pyridazine), has been produced successfully through applying a mixed ligand synthesis method via reacting Ni(NO_3_)_2_·6H_2_O with 3,6-bis(benzimidazol-1-yl)pyridazine ligand in the presence of a carboxylic acid co-ligand. Hyaluronic acid (HA) and carboxymethyl chitosan (CMCS) are representatives of natural polysaccharides and have good biocompatibility. Based on the chemical synthesis method, HA/CMCS hydrogel was successfully prepared. SEM showed that the lyophilized gel presented a typical macroporous structure with three-dimensional connected pores, which had unique advantages as a drug carrier. Using paclitaxel as a drug model, we further synthesized a novel paclitaxel-loaded metal gel and evaluated its therapeutic effect on cervical cancer. Finally, novel drugs from the reinforcement learning simulation are suggested to have better biological activity against ovarian cancer due to low affinity energy and stronger interaction strength towards the protein receptor.

## Introduction

Cervical cancer is a common malignant tumor of the female reproductive system, with the 4th highest morbidity and mortality rate in the world, and the morbidity and mortality rates are on the rise, and gradually becoming younger, seriously threatening women’s health^1^. Clinical studies have found that the treatment effect of early-stage cervical cancer patients is remarkable, but the treatment effect and prognosis of middle- and late-stage cervical cancer patients are unsatisfactory. Therefore, searching for new therapeutic targets for cervical cancer has become a new trend and a hotspot for research^2^. Hepatocyte nuclear factor 1A (HNF1A) is involved in tumor cell proliferation and growth, and induces epithelial mesenchymal transition. HNF1A plays a key role in tumorigenesis and tumor development and is highly expressed in cervical cancer patients, which can promote tumor cell proliferation and shorten the survival period of cervical cancer patients.

Coordination chemistry and supramolecular chemistry are now interested in the structure together with design of metal-involved supramolecular structures on the basis of crystal engineering^3–5^. Their remarkable unit structure and possible applications in biochemistry, luminescence as well as catalysis—particularly in contemporary medicinal chemistry—justify the growing attention in this topic^3,6,7^. Several synthetically derived functional complexes with catalytic, polymeric, and bioactive properties have been reported. Explorations into various anticipated medicinal applications have been conducted using density functional theory, computer predictions, and molecular modeling, generating significant interest^8–12^. Therefore, choosing biocompatible, effective, and safe ligands has become essential in the therapeutic applications, pharmacological treatment together with structural design. Polydentate ligands, encompassing polycarboxylic acids or heterocyclic ligands containing nitrogen, are extensively employed in the methodical design and production of these multifunctional CPs. Derivatives of benzothiadiazole having an electron-deficient and π-conjugated group that have outstanding optical and biological qualities^13–16^. The L ligand is a semi-rigid imidazole ligand that possesses a possible unsaturated site. This property helps it create a contact with target proteins, potentially producing CPs with intriguing bioactivity^17–22^. In this work, a novel CP based on Ni(II) has been produced successfully through applying a mixed ligand synthesis method via reacting Ni(NO_3_)_2_·6H_2_O with L ligand in the presence of a carboxylic acid co-ligand.

Paclitaxel was first extracted from taxus chinensis in 1967 and was approved by the FDA for clinical cancer treatment in 1992^23^. Since then, paclitaxel has become the first line of chemotherapy for many cancers, including ovarian cancer, non-small cell lung cancer, and cervical cancer^24–26^. Paclitaxel can specifically bind to β tubulin, inhibit microtubule depolymerization and inhibit tumor cell proliferation. However, the traditional drug delivery method has some shortcomings, such as poor water solubility, short half-life and little accumulation at the tumor site, which seriously affect the therapeutic effect of chemotherapy^27^. Hydrogel has good biocompatibility and biodegradability, and is combined with drugs by physical adsorption or chemical adsorption to make corresponding dosage forms, and enters the human body through diffusion, osmosis and other ways, so that drug molecules can be released slowly and continuously in the human body at a stable and controllable rate and appropriate concentration, so as to achieve the purpose of giving full play to the drug effect and effectively treating diseases^28,29^. Therefore, hydrogels have unique advantages as drug carriers and have been widely concerned by researchers.

In this study, we designed and synthesized paclitaxel-loaded metal gel and investigated their role in the treatment of cervical cancer. After treating cervical cancer cells with different concentrations of the drug, HNF1A, the main marker gene of cervical cancer, was significantly down-regulated with the increase of drug concentration. The results showed that the paclitaxel-loaded metal gel inhibited the proliferation of cervical cancer cells by targeting and suppressing the expression of HNF1A.

Finally, the traditional drug compound design and development are time and effort consuming, the machine learning technic has been proved to have capability in performing high-throughput drug molecule screening and therefore to provide an efficient way for design and discover novel drug compounds. Along with the experiment study, machine learning simulation has been carried out to design and develop novel anti-cancer drug compounds using the experimental synthesized compound as the lead structure.

## Experimental

### Chemicals and measurements

All of the reagents and solvents are marketable and do not require further purification. Through Thermo Flash EA 1112-NCHS-O analyzer, the EA of N, H and C was implemented. IR spectra were derived via applying a Brucker Equinox-55 FT-IR spectrophotometer as KBr pellets.

Hyaluronic acid (HA) carboxymethyl chitosan (CMCS), 1-ethyl-3-(3-dimethylaminopropyl) carbodiimide (EDC), N-hydroxysuccinimide (NHS) were purchased from Sinopod Chemical Reagents Co., Ltd.

### *Preparation and characterization for [Ni(L)(D-CAM)(H*_*2*_*O)]*_*n*_* (1)*

The mixture composed of 0.1 mmol and 30.0 mg Ni(NO_3_)_2_·6H_2_O, 31.2 mg and 0.1 mmol L, 20.0 mg and 0.1 mmol D-H_2_CAM, HBF_4_ (8 drops, 37% aq) in 8 mL of H_2_O were heated in a autoclave (25 mL) lined with Teflon at 120 °C for 12 h, and later cooled to ambient temperature. **1**’s green nubby crystals were gathered and rinsed utilizing methanol and water. Yield: about 42% (on the basis of L). Elemental analysis (%): Anal. Calcd for C_28_H_28_N_6_O_5_Ni: N, 14.30; H, 4.80; C, 57.24. Found: N, 14.14; H, 4.75; C, 56.62. IR (cm^−1^, KBr pellets): 619(m), 731(s), 762(m), 913(w), 976(w), 1196(m), 1220(m), 1254(m), 1283(m), 1313(s), 1356(s), 1382(s), 1438(s), 1455(s), 1503(m), 1529(m), 1566(s), 1910(w), 2360(w), 2881(w), 2960(w), 3127(w).

XRD data was derived from SuperNova diffractometer and intense data were investigated with CrysAlisPro software and subsequently transformed into HKL files. The creation together with refinement of initial model of construction were implemented via the SHELXS program utilizing direct approach along with SHELXL-2014 program through least-squares approach. Anonymous parameters can be mixed following adding entire non-H-atoms. Ultimately, the whole H-atoms could be fixed to geometrically linked C-atoms by means of the AFIX command. CPs’ refinement details along with their crystallographic parameters were presented in Table 1.

**Table 1:** CPs’ refinement details and their crystallographic parameters.

Empirical formula	C_28_H_28_N_6_NiO_5_
Formula weight	587.27
Temperature/(K)	293(2)
Crystal system	monoclinic
Space group	C2/c
a/(Å)	17.6327(2)
b/(Å)	20.7419(5)
c/(Å)	13.55280(10)
α/(°)	90
β/(°)	95.693(2)
γ/(°)	90
Volume/(Å^3^)	4932.30(14)
Z	8
ρ_calc_g/(cm^3^)	1.582
μ/(mm^−1^)	0.842
Reflections collected	16772
Independent reflections	6091 [R_int_ = 0.0328, R_sigma_ = 0.0429]
Data/restraints/parameters	6091/7/365
Goodness-of-fit on F^2^	1.093
Final R indexes [I > = 2σ (I)]	R_1_ = 0.0829, ωR_2_ = 0.2086
Final R indexes (all data)	R_1_ = 0.0933, ωR_2_ = 0.2156
Largest diff. peak/hole/(e Å^−3^)	2.58/− 0.96
CCDC	2333456

### Preparation and characterization for paclitaxel-loaded metal gel

Firstly, 1 g HA powder and 3 g CMCS powder were weighed and dissolved in 100 ml of deionized water, respectively. The EDC/NHS solution was slowly dropwise added to the HA solution and stirred rapidly for 30 min. Then, the HA mixture and CMCS solution were mixed according to the same volume and quickly stirred before being added to the mold. The HA/CMCS hydrogel was prepared by standing at 4 ℃ overnight and washing with deionized water after gel molding. The Ni(II) comlex was immersed in 10 mg/ml paclitaxel solution for ultrasonic dispersion and then added to CMCS solution to prepare the paclitaxel-loaded metal gel.

The microscopic morphology of the sample was observed by scanning electron microscope. Before the test, the sample was freeze-dried and sprayed with gold.

### Realtime PCR

The cervical carcinoma cell line SiHa was cultured in 24-well plate and treated with different concentrations of paclitaxel-loaded metal gel for 48 h. The RNA was isolated with Trizol reagent (Invitrogen, USA). The RNA was reverse transcript into cDNA by iScript^™^ cDNA Synthesis Kit (BioRad, USA). The Realtime PCR was performed on the ABI 7500 Fast Reatime PCR system (ABI, USA). The relative expression of HNF1A was calculated by 2^−∆∆Ct^ method.

### Simulation methods

In this work, a framework called molecule deep Q-networks (MolDQN)^30^ has been utilized for the optimization of the molecules using the reinforcement learning approach. Practically speaking, the optimization of a molecule based on the given template contains four types of actions, namely, atom addition, bond addition, bond removal and so called “no modification”. During the optimization, how far away a molecule can go from the given template is controlled by the limit of number of steps, which is set to 16. The total number of optimized molecules is set to 6000. As the number of the optimized molecule increasing, the abovementioned actions would change from a random-based selection to a reward-based selection, such a procedure is called ε-greedy decaying, where the initial value of ε is 1.0, indicating a fully random selection of the actions, the decrement of ε is 1/6000, so the preference of the action selection gradually increases. In such a manner, the latter generated molecule tends to have relatively high rewards.

Three types of rewards are adopted for the molecule optimization using the reinforcement learning approach, i.e., the binding affinity between the protein receptor and optimal molecule, the synthetic accessibility (SA) score, and the quantitative estimate of drug-likeness (QED) score. The binding affinity is calculated by QuickVina 2, which has been proven to have both accuracy and efficiency. The SA score will be evaluated through gym-molecule library. The QED score will be determined using rdkit (https://www.rdkit.org/). For these three evaluations, the general input is the Smile code of the molecular structure, and during the molecular docking, the 3-dimensional structure was generated by open babel.

To explore the CP’s bio-activity, including the synthesized compound and the optimized compounds, to the cervical cancer, the estrogen receptor has been used as the receptor protein for the molecular docking simulation, since its overexpression has the direct relationship to the cervical cancer. The protein structure of the estrogen receptor was obtained by downloading from the Protein Data Bank with a PDB ID of 1ERE^31^. On the other hand, the compound that was used as the lead structure for the reinforcement learning was taken from the synthesized Ni complex. The dimension of the grid box is 80 × 60 × 60 in three directions, such a grid box is large enough to contain the docking pocket of interest^32,33^. The distance between the grid points is 0.375 Å. The center of the grid box is at 23.895, 49.094, 134.275 Å. After the reinforcement learning process, the outstanding optimized compound was coordinated to the Ni ion with the co-ligand, and the new complexes were evaluated by AutoDock 4 with the Lamarckian Genetic Algorithm (LGA), the number of binding poses was 20.

## Results and discussion

### Crystal structure description

**Figure 1:**
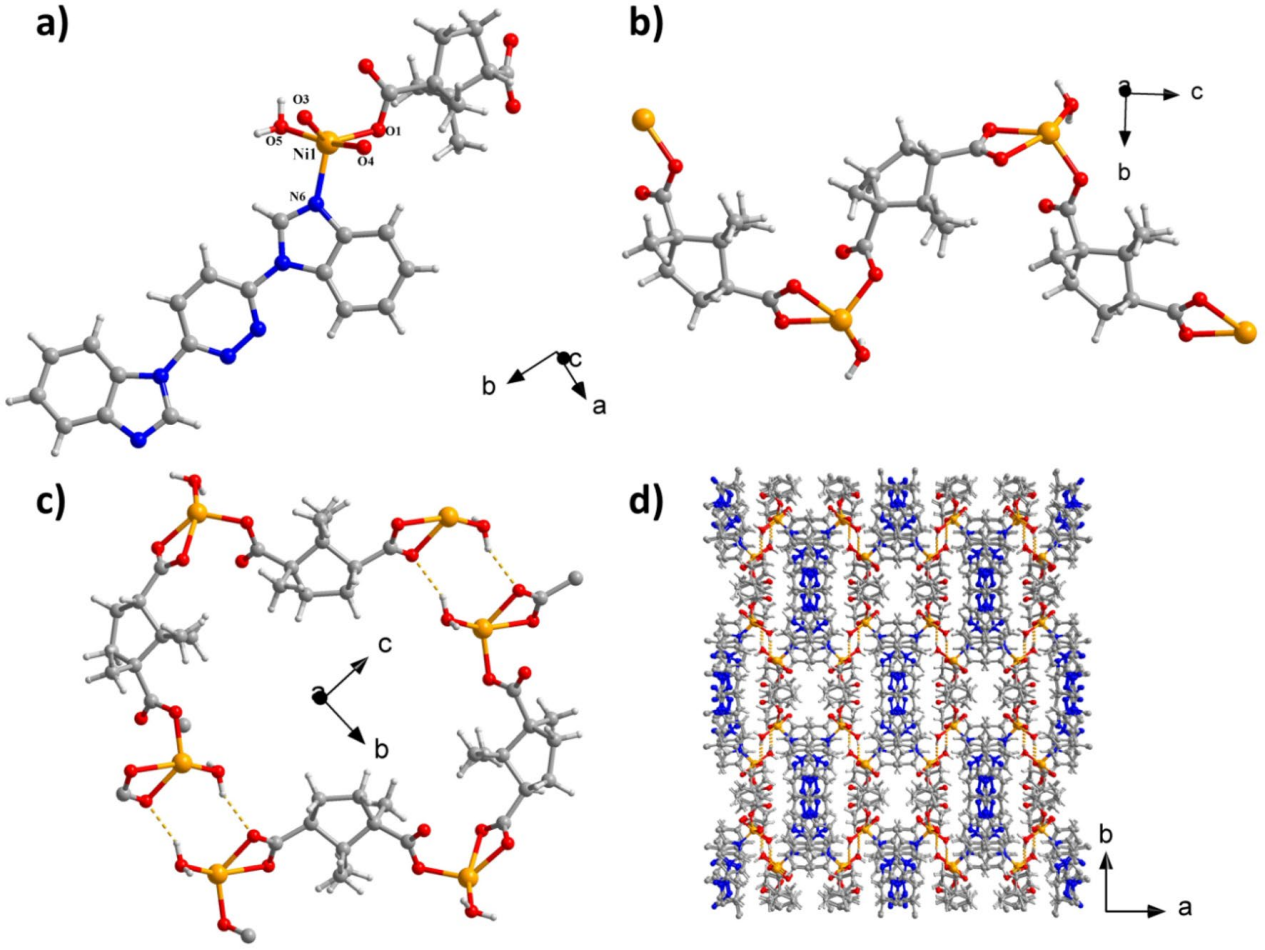
(**a**) **1**’s asymmetry unit. (**b**) The 1-dimensional double-chain structure. (**c**) The H-bon interactions between neighboring chains. (**d**) **1**’s 3D H-bond supramolecular network.

Structural analysis indicates that CP **1** was crystallized in the monoclinic space group *of C*2/*c* and possesses a 3D supermolecular structure. The unsymmetric unit is composed of a L ligand, an independent Ni^II^ ion, a coordination molecule of water, as well as a deprotonation ligand of D-CAM^2−^. The Ni1 ion has five ligands and is coordinated by O1, O3, O4, and O5 atoms derived from two distinct ligands of D-CAM^2−^ and a coordinating molecule of water, as well as a N6 atom from a separate L ligand. Analysis of the coordination geometry surrounding the Ni^II^ center revealed that the Ni^II^ ion is in a twisted pentahedral setting (Fig. 1a). The Ni–N and Ni–O bond has a length of 2.031(4) Å and 1.932(4)–2.308(4) Å. Ni1 atoms and coordinate atoms are located at the center and apex of the pentahedron, respectively. The neighboring nickel center was bridged with the carboxyl oxygen atom of the D-CAM^2−^ ligand to create an infinitely extended one-dimensional interpenetrating double-stranded structure (Fig. 1b) with a Ni–Ni spacing of 9.502 Å. Neighboring 1D chains are connected through H bonds (O5-H5B…O3) between the carboxyl O atoms and coordinated molecules of water to create a 2D layered Ni − organic network (Fig. 1c). Lastly, the 2D network develops a 3D supramolecular architecture through another H bonds (O5-H5A…N1) between the imidazole N atoms and coordinated molecules of water (Fig. 1d). In the three-dimensional supramolecular structure, the imidazole N-atoms are in a single-coordination pattern and D-CAM^2−^ ligand is in a (*κ*^1^)-(*κ*^2^)-*μ*_2_ coordination pattern.

### Micromorphology of the hydrogels

**Figure 2:**
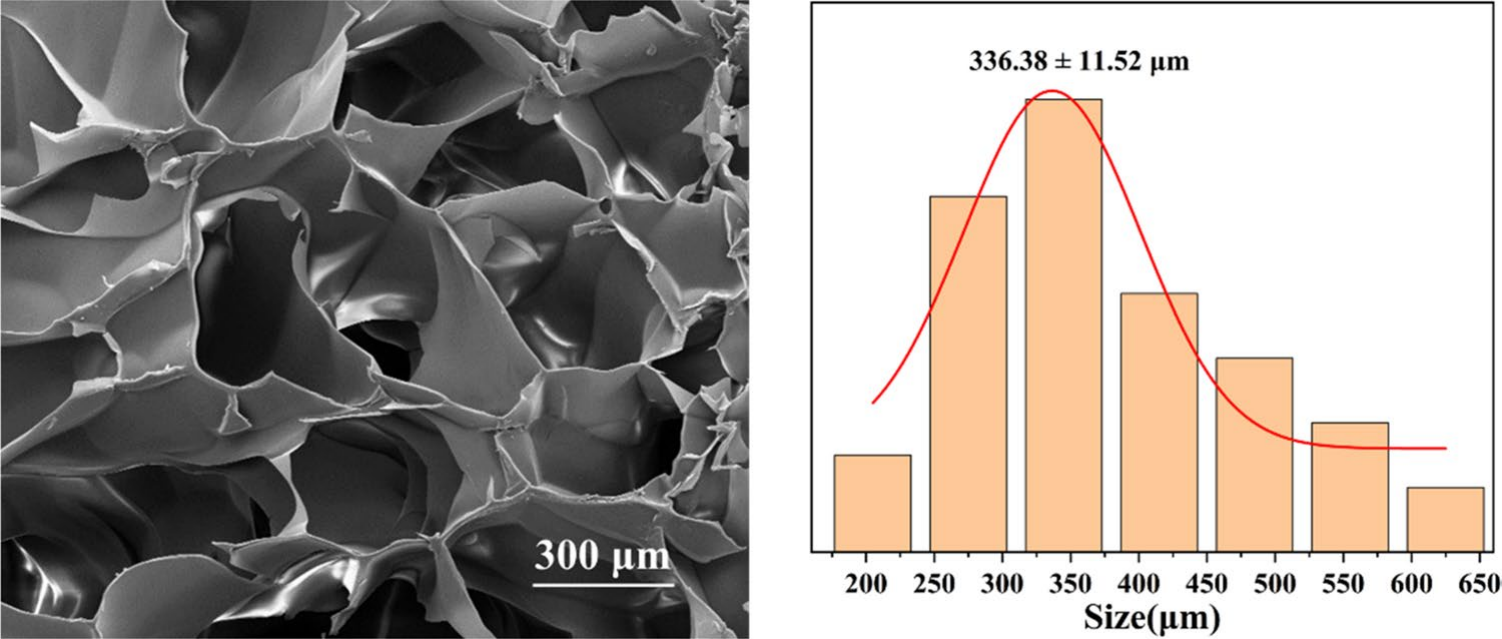
Microstructure and pore size distribution of HA/CMCS hydrogels.

Hydrogels are three-dimensional network structures formed by physical or chemical crosslinking of hydrophilic polymers, which have good biocompatibility and ion transport ability, and are often used as drug delivery carriers and medical dressings. Because of its good hydrophilicity, stable porous structure and good fluidity, it is easy to transport drugs, and it is more targeted than ordinary drugs to treat diseases in specific parts. The internal microstructure of HA/CMCS hydrogel was observed by scanning electron microscope after freeze-drying. As shown in Fig. 2, the freeze-dried HA/CMCS gel showed a typical large-pore structure with interconnected pores and a concentrated pore size distribution of 336.38 ± 11.52 μm.

### Paclitaxel-loaded metal gel inhibits the expression of HNF1A

**Figure 3:**
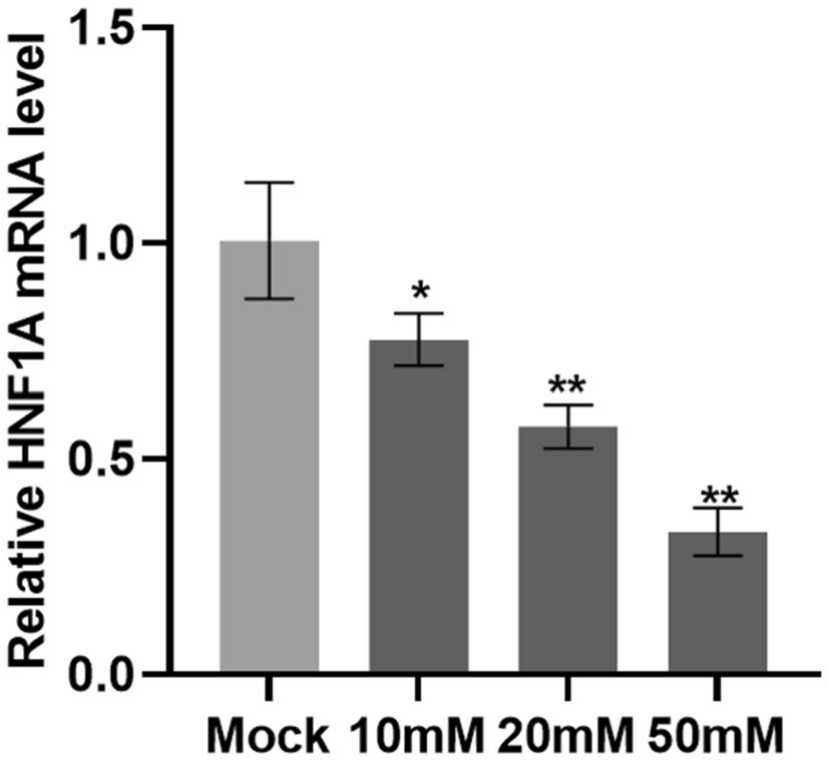
Relative expression of HNF1A in SiHa cells after the treatment of paclitaxel-loaded metal gel. Single asterisk and double asterisk indicated P < 0.05 and 0.01, respectively.

Since HNF1A expression is upregulated in cervical cancer cells. To investigate whether paclitaxel-loaded metal gel could inhibit the expression of HNF1A. Cervical cancer cell line SiHa was treated with different concentrations of paclitaxel-loaded metal gel for 48 h, and the mRNA level of HNF1A was detected by Realtime PCR. As shown in Fig. 3, the mRNA level of HNF1A was significantly down-regulated after drug treatment in a dose-dependent manner, and the expression of HNF1A was weaker with increasing drug concentration. Our results suggest that paclitaxel-loaded metal gel may have inhibited the proliferation of cervical cancer lineage cells by down-regulating HNF1A.

### Reinforcement learning predictions and molecular docking simulations

It has been demonstrated that the growth, progression, as well as homeostasis of a variety of tissues are directly linked to estrogen receptors^26^, and estrogen receptor overexpression has a major role in the genesis of cervical cancer. Thus, the inhibition of the overexpression of estrogen receptor can be viewed as an effective characteristic for evaluating the bio-activity of drug compound against cervical cancer. The aforementioned experiments have confirmed the bio-activity of Ni compound against cervical cancer, but the development of such drug compound is nevertheless time and effort consuming. Machine learning technic can perform high-throughput drug molecule screening and therefore provide an efficient way for design and discover novel drug compounds. Using the experiment verified compound as the lead structure, 6000 episodes were optimized with the aim of developing novel structures that have the potential of anti-cancer activity.

**Figure 4:**
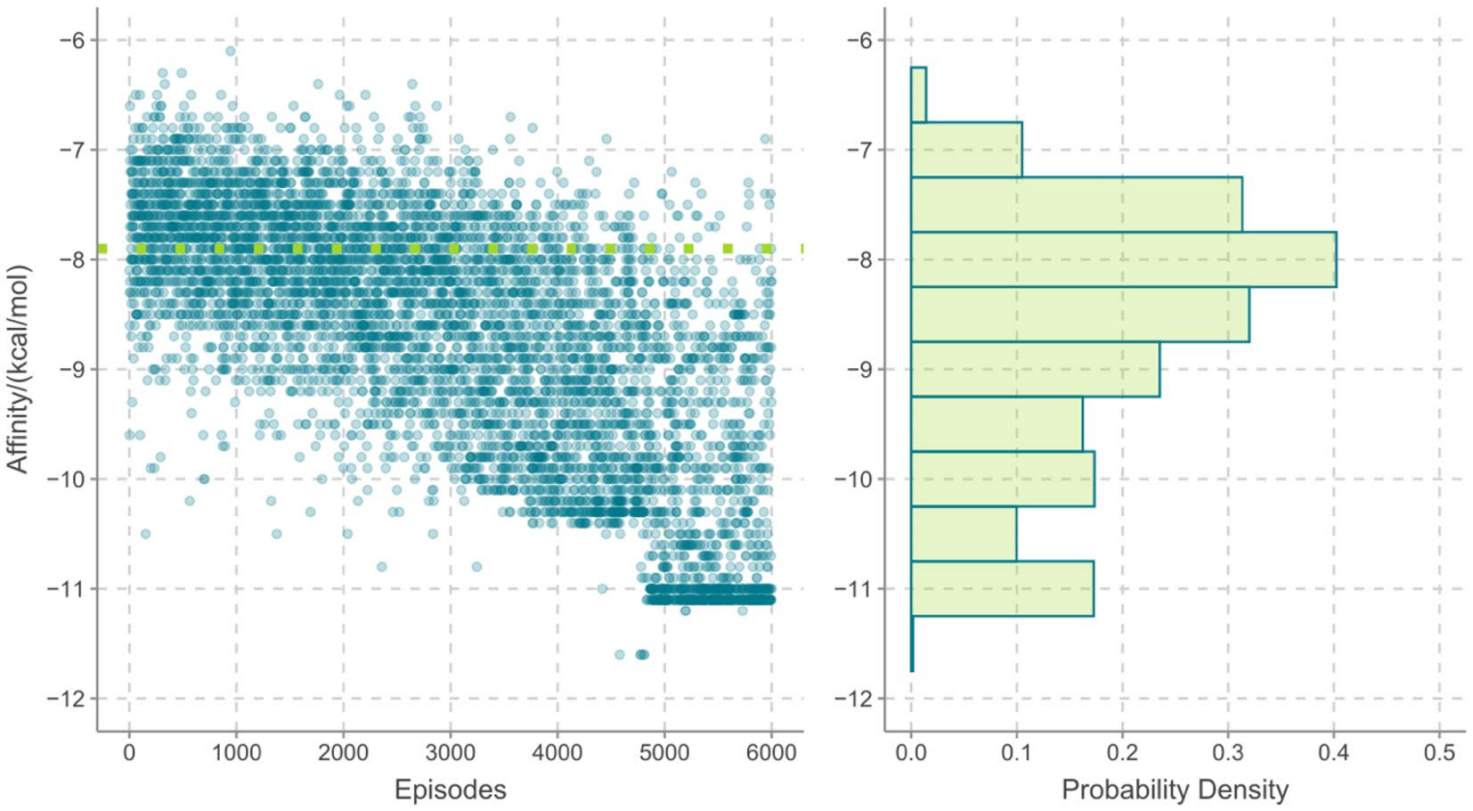
The affinity energy and the energy probability density for 6000 episodes, the dotted line indicates the value for the lead structure.

In Fig. 4, the affinity energy of 6000 episodes as well as the probability density are shown. Firstly, we can see that the overall trend for the affinity energy decreases with increasing number of optimized episodes, such a trend validates the success of the ε-greedy decaying method, meaning the optimizing direction should focus on modifying new episode with lower binding affinity. Further, we can see that the episodes with an affinity of − 8.0 kcal/mol have the highest probability. And more and more optimized episodes are better, at least for the affinity energy, than the lead structure.

**Figure 5:**
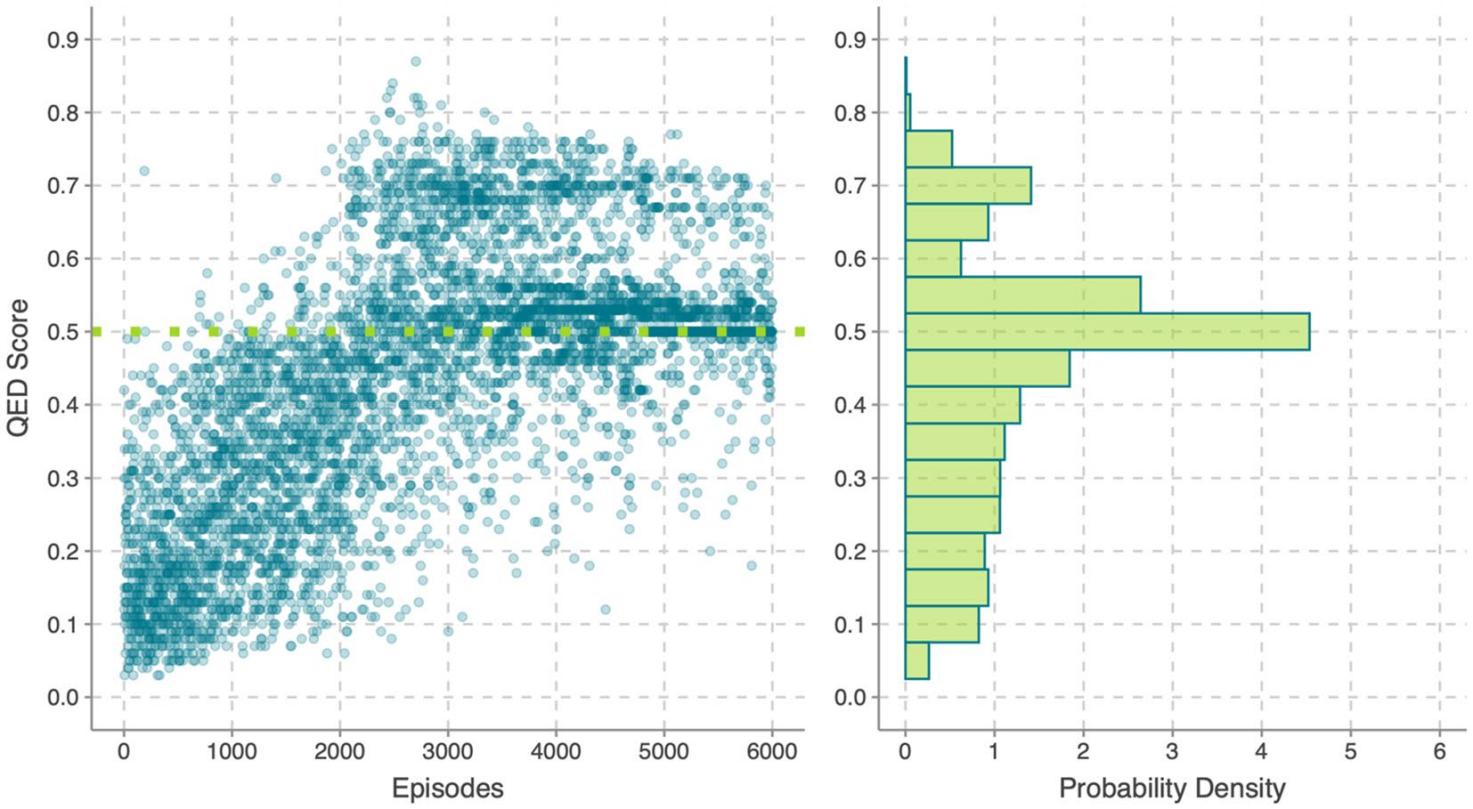
The QED score and the score probability density for 6000 episodes, the dotted line indicates the value for the lead structure.

The QED score and its probability density are shown in Fig. 5. Similar to the results for affinity energy, the QED score increases with increasing number of optimized episodes. The highest probability is seen to occur at 0.5, which is also the QED score for the lead structure. The QED score seem to converge at two discrete positions, meaning around 0.5 and 0.7, respectively, and a small peak is seen at 0.7. There is a broader shoulder from 0.1 to 0.4, indicating a considerable fraction of episodes may not be effective for their low QED scores.

**Figure 6:**
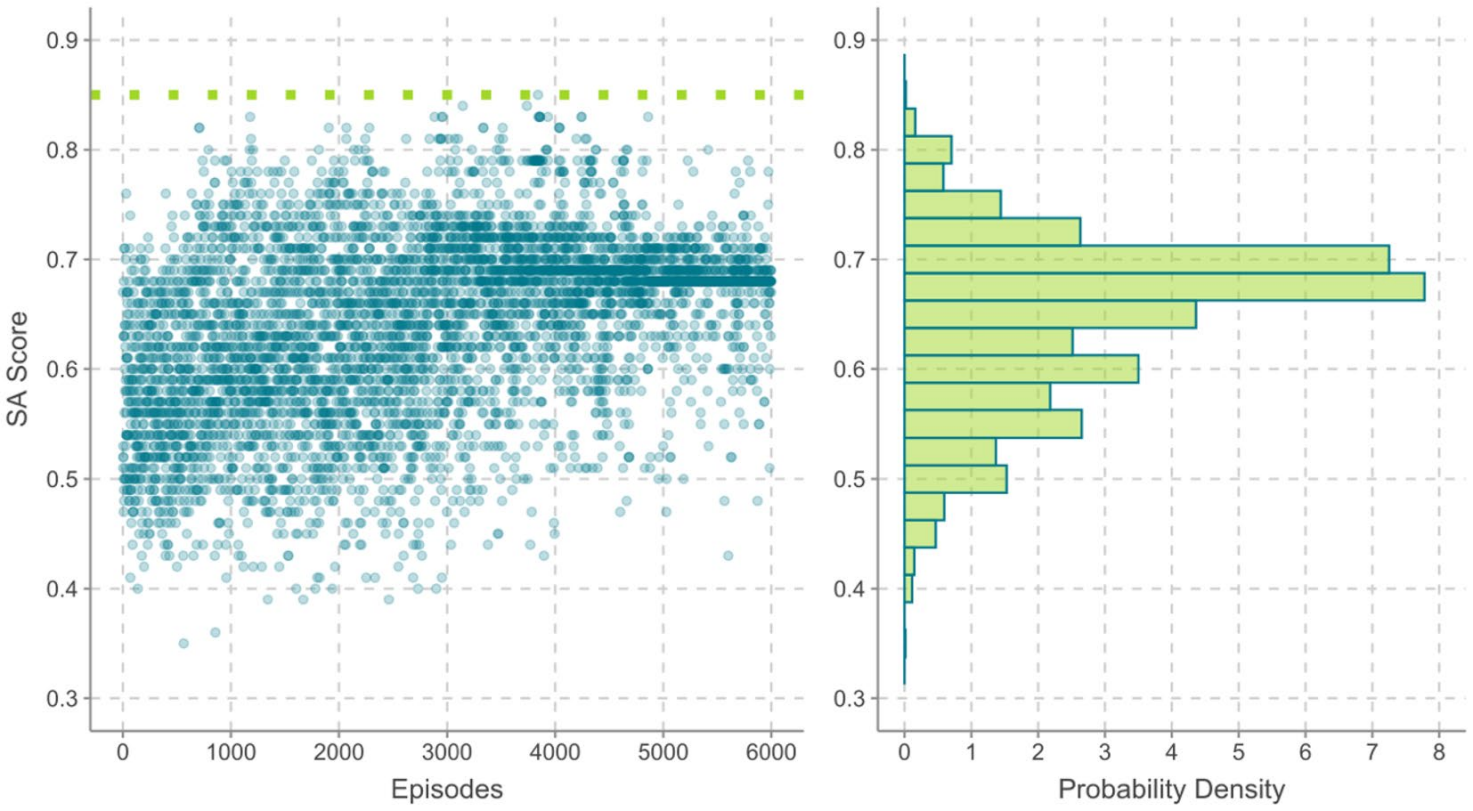
The SA score and the score probability density for 6000 episodes, the dotted line indicates the value for the lead structure.

The third reward is the SA score, which is shown in Fig. 6. From the dotted line we can see that the SA score for the lead structure sets an upper bound at 0.85, suggesting that the lead structure is easy to synthesize in reality. Although the optimized episodes cannot have a competitive SA score as the lead structure, but the SA score trend is increasing with increase in the number of optimized episodes, and the SA score is seen to converge near 0.7, which contributes the highest probability.

**Scheme 1.**
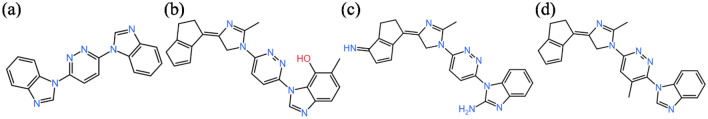
(**a**) The lead structure used in the reinforcement learning simulation, (**b**) the optimized structure with its affinity, QED and SA scores of − 10.3 kcal/mol, 0.70 and 0.68, (**c**) the optimized structure with its affinity, QED and SA scores of − 10.2 kcal/mol, 0.71 and 0.67, and (**d**) the optimized structure with its affinity, QED and SA scores of − 10.3 kcal/mol, 0.71 and 0.63.

For screening out the drug compounds with higher probability with potential biological activity against the cervical cancer, we would like to select structures with high SA and QED scores and low affinity energy, based on the above results and the probability densities, the thresholds for SA, QED and affinity scores are 0.6, 0.7 and − 10.0 kcal/mol. These thresholds individually guarantee that approximately 18.2%, 22.6% and 12.3% structures from 6000 episodes have better values than the other part of episodes. In the end, we choose three representative structures which satisfy all three thresholds for further analysis, these structures as well as the lead structure are shown in Scheme 1.

**Figure 7:**
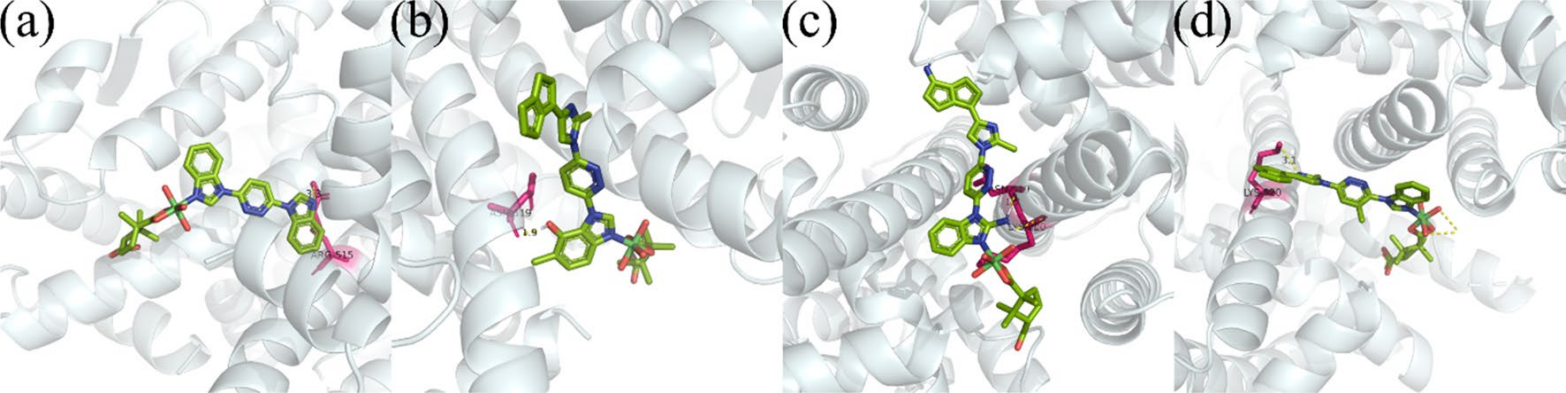
Binding poses for the protein receptor 1ERE with (**a**) the Ni complex from the experiment, the affinity energy is − 6.55 kcal/mol, (**b**) the optimized compound with an affinity of − 9.13 kcal/mol, (**c**) the optimized compound with an affinity of − 11.19 kcal/mol, and (**d**) the optimized compound with an affinity of − 7.45 kcal/mol.

To further understand the inhibition effect of the compounds, the structures in Scheme 1 are coordinated to the Ni ion and have been studied in detail from molecular scale. The binding poses between the protein receptor and compounds are shown in Fig. 7. From Scheme 1, one can conclude that the active functional groups are the existing imidazole group, the newly generated “–OH”, “ =NH” and “ =N–” groups. Hence, the binding interactions should be formed from these groups, which can be confirmed from the binding poses. Explicitly, in Fig. 7a, the imidazole ring interacts with residue ARG-515, the affinity energy is − 6.55 kcal/mol, and the hydrogen bond length is 3.3 Å, such a distance is normally viewed as a moderate interaction. In Fig. 7b, the hydrogen bond is forming between the “–OH” and residue ASN-519, the bond length and affinity energy is 1.9 Å and − 9.13 kcal/mol, respectively. Figure 7c presents that two H bonds are produced by the “ =NH” group with residues ASN-519 (1.7 Å) and LYS-520 (2.4 Å), and the affinity energy is − 11.19 kcal/mol. In Fig. 7d, one hydrogen bond has formed between the imidazole group and residue LYS-520, the bond length is 3.3 Å and the affinity is − 7.45 kcal/mol. The above results suggest that two of optimized compounds may have better biological activity than the lead structure from based on the lower affinity energy and stronger hydrogen bond lengths. In contrast, the rest optimized compound has comparable biological activity as the lead structure, due to similarity in both affinity and hydrogen bond length. Moreover, based on the above results, the reinforcement learning simulation has been proved to be an effective and efficient method for screening novel drug compounds.

## Conclusion

Taken together, we have succeeded in producing a new Ni(II)-based CP through reacting Co(NO_3_)_2_·6H_2_O with the L ligand in the presence of carboxylic acid co-ligand. The structure and chemical composition were studied by SCXRD, IR spectra, and EA. In addition, HA/CMCS hydrogels were prepared from hyaluronic acid and carboxymethyl chitosan by chemical synthesis method. The SEM results showed that the freeze-dried gel showed a three-dimensional porous network structure with interconnected pores and a concentrated pore size distribution of 336.38 ± 11.52 μm, which was an ideal drug carrier. Using paclitaxel as a drug model, we further designed and synthesized paclitaxel-loaded metal gel and studied its role in the treatment of cervical cancer. Biological results showed that the mRNA level of HNF1A was significantly down-regulated in SiHa cells after paclitaxel-loaded metal gel treatment in a dose-dependent manner. Paclitaxel-loaded metal gel may have inhibited the development of cervical cancer through down-regulation of HNF1A, and it is expected to be developed as a novel drug for the treatment of cervical cancer. A high-throughput machine learning framework based on the deep Q-networks has been performed for optimizing novel compounds that have potential biological activity against cervical cancer. The optimized compounds have been further validated by the molecular docking simulation and the results suggest that those compounds have even better biological activity because of lower affinity energy and stronger binding strength towards the receptor.

## Data Availability

The article contains the data utilized to support the results of the research.
